# Stable and Controllable Synthesis of Silver Nanowires for Transparent Conducting Film

**DOI:** 10.1186/s11671-017-1963-6

**Published:** 2017-03-23

**Authors:** Bitao Liu, Hengqing Yan, Shanyong Chen, Youwei Guan, Guoguo Wu, Rong Jin, Lu Li

**Affiliations:** 1 Research Institute for New Materials Technology, Chongqing University of Arts and Sciences, Yongchuan, Chongqing, 402160 China; 2 School of Mechatronics Engineering, Chongqing University of Arts and Sciences, Yongchuan, Chongqing, 402160 China; 3 Chongqing Co-Innovation Center for Micro/Nano Optoelectronic Materials and Devices, Chongqing University of Arts and Sciences, Yongchuan, Chongqing, 402160 China

**Keywords:** Silver nanowires, Without particles, Transmittance, Conductive

## Abstract

Silver nanowires without particles are synthesized by a solvothermal method at temperature 150 °C. Silver nanowires are prepared via a reducing agent of glycerol and a capping agent of polyvinylpyrrolidone (*M*_*w*_ ≈ 1,300,000). Both of them can improve the purity of the as-prepared silver nanowires. With controllable shapes and sizes, silver nanowires are grown continuously up to 10–20 μm in length with 40–50 nm in diameter. To improve the yield of silver nanowires, the different concentrations of AgNO_3_ synthesis silver nanowires are discussed. The characterizations of the synthesized silver nanowires are analyzed by UV-visible absorption spectroscopy, X-ray diffraction (XRD), scanning electron microscopy (SEM), and atomic force microscope (AFM), and silver nanowires are pumped on the cellulose membrane and heated stress on the PET. Then, the cellulose membrane is dissolved by the steam of acetone to prepare flexible transparent conducting thin film, which is detected 89.9 of transmittance and 58 Ω/□. Additionally, there is a close loop connected by the thin film, a blue LED, a pair of batteries, and a number of wires, to determinate directly the film if conductive or not.

## Background

Because of optical, electrical, and thermal properties, silver nanowires are widely studied and used in many applications like transparent electrodes, plasmonic antennae for surface-enhanced Raman scattering (SERS), optical polarizers, biomolecular sensor, catalysts, and batteries in recent years [1–4]. For these broaden applications, the synthesis of Ag nanowires has attracted the general scholars in the past decades. Through scientists constantly efforts, it has accumulated a lot of methods, such as template method, self-assembly method, hydrothermal synthesis method, and polyol method, to synthesis different length to diameter ratio of silver nanowires. But the most commonly used preparation method is the polyol method. Younan Xia and his partners [5] originally synthesized Ag nanowires with nanodiameters through a polyol reduction process with assistance of Pt nuclei. Silver was reduced on (111) face of Pt and grown to silver nanowires along <110> by polyvinylpyrrolidone (PVP) as dispersant and capping agent. Later, Younan Xia team reported more than ten papers related to silver nanowires synthesis. Then on that basis, other metal halide control agents and non-metal halide control agents, for instance NaCl [6, 7], FeCl_2_ or FeCl_3_ [8], CuCl_2_ [9], TPA-B, and TPA-C [10], etc, have been developed to improve effectively the quality of the Ag nanowires by selective ecting with the presence of halide ions. As a consequence, the abovementioned methods have been regarded as the most promising method to synthesize silver nanowires with high aspect ratios. Stephen M. Bergin and co-workers [11] prepared different length and diameter of silver nanowires, which were discussed the influence of transparency and electrical conductivity of transparent film prepared by silver nanowires. However, glycerol as a sole reducing agent on synthesis of silver nanowires has been rarely reported. Changchao Jia et al. [12] prepared uniform multiple crystalline silver nanowires by glycerol and ethylene glycol co-mediated and discussed the effect of different volume ratios of EG and glycerol on the synthesis of Ag NWs. L.R. Shobina and other teams [13] synthesized silver nanowires by glycerol as reducing agent instead of ethylene glycol in the round bottom flask.

Indium tin oxide (ITO) thin film can show as high as 95% transmittance (*T*) with resistance (*Rs*) <100 Ω/□, but the expensive cost, emerging indium scarcity and brittle property of ITO, results to be limited to apply in flexible film [14]. However, silver has the highest electrical conductivity (6.3 × 107 S/m) and thermal conductivity (429 W m^−1^ K^-1^) among all the metals [15]. Due to surface-enhanced Raman scattering (SERS) and optical polarizers, silver nanowires are considered as very promising candidates in flexible electronics. As a consequence of the abovementioned drawbacks of ITO applied in thin film, silver nanowires prepared transparent conductive thin film became possible. Besides, silver nanowires have large aspect ratios to prepare flexible transparent conductive thin film with low sheet resistance, and the silver nanowires have other applications, such as transparent electrode and transparent film heater. QJ Huang reported AZO/AgNWs/AZO deposited onto polyimide films, which displays excellent thermostability and mechanical flexibility with the low surface roughness (*R*-rms < 8 nm). Huang et al. [16, 17] reported silver nanowires successfully embedded in polyimide films to prepare highly flexible and transparent film heaters.

The size of Ag particles synthesized in the process of silver nanowire preparation is usually large. The particles are not completely removed by centrifugation. Even though using density gradient centrifugation, a small quantity of nanoparticles exists in silver nanowires and a lot of nanowires are lost. Therefore, the roughness and optical properties of silver nanowire films are affected, and the conductive and optical properties of the other particles of inoculating seed such as Pt, Fe, and Cu, are lower than Ag. In this paper, the synthesis of silver nanowires without nanoparticles and flexible transparent conducting thin film prepared is reported. In this typical synthesis process, silver nanowires are prepared by a modified traditional polyol process where glycerol is prepared instead of ethylene glycol in the presence of PVP and glycerol without other agents in the autoclave. Remarkably, the process is very simple to put mixed solution in autoclave one step at room temperature, and the obtained silver nanowires have no other particles and impurities. Thus, silver nanowires are successfully dispersed in the isopropyl alcohol after the purification. Then, firstly, the above silver nanowires are pumped on the cellulose membrane, which is pasted on the PET by the reciprocating rolling. After cellulose membrane was dissolved, the flexible transparent conducting thin film is prepared completely. Uniform silver nanowires can contribute to the stability of flexible transparent conducting thin film. In the end, the properties of the synthesized silver nanowires and the flexible transparent conducting thin film are demonstrated and the results are presented.

## Methods

### Reagents

AgNO_3_ and glycerol were purchased from Chengdu Kelong Chemical Reagent Factory. Polyvinylpyrrolidone (PVP) (weight-average molecular weight, *M*_*w*_ ≈ 1,300,000) was purchased from Sigma-Aldrich. All above, the reagents were AR grade.

### Experimental Procedure

#### Preparation of Silver Nanowires

**Fig. 1:**
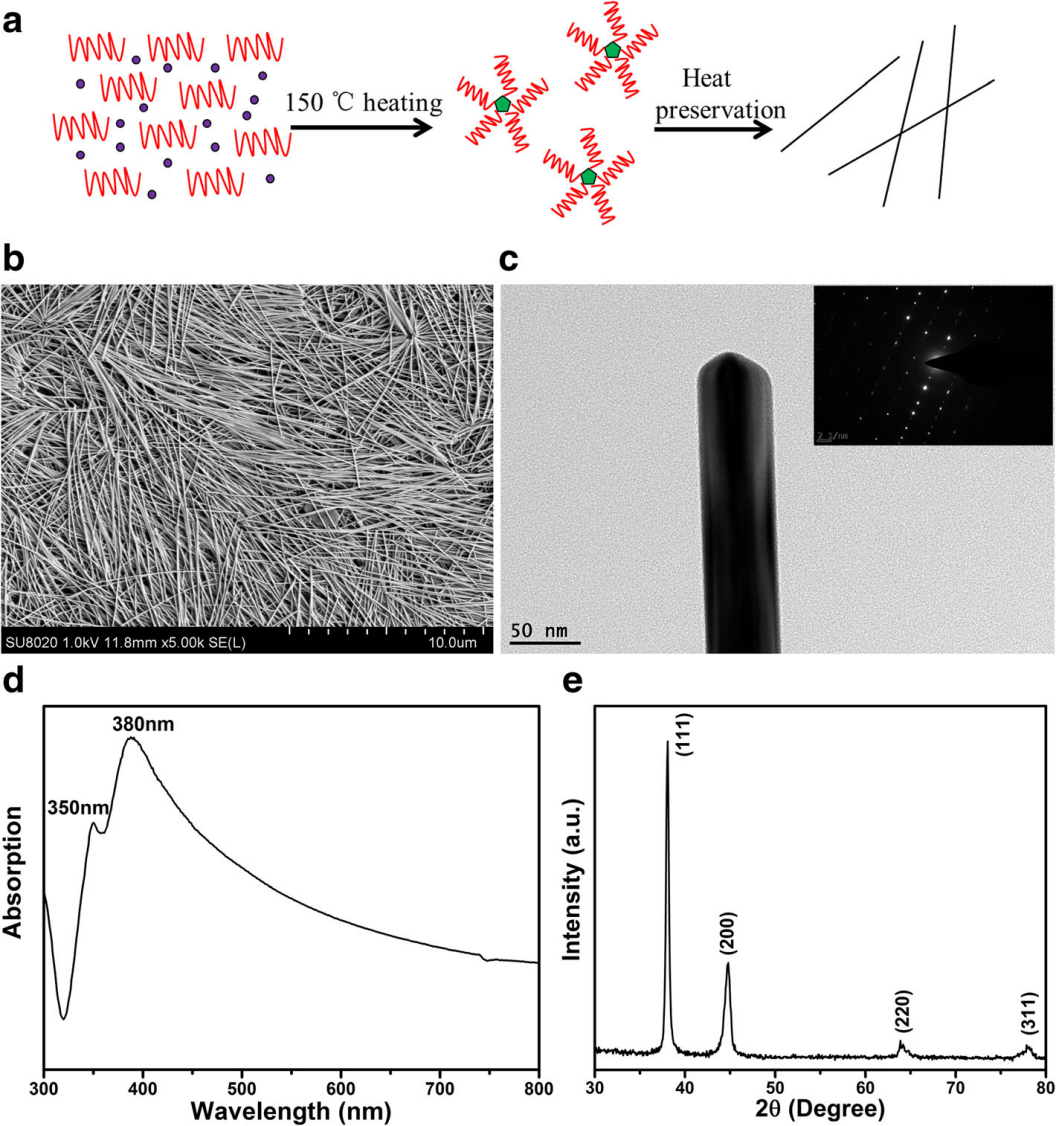
The synthesis process of silver nanowires (**a**), SEM (**b**), TEM (**c**), UV-vis-NIR absorption spectra (**d**), and XRD pattern of silver nanowires (**e**)

Ethylene glycol as a common polyol solution has been widely used to reduce Ag^+^ in the process of synthesis of silver nanowires. But to synthesize thin and uniform silver nanowires, glycerol as a solvent and reducing agent is more stable for silver nanowire synthesis. Glycerol has three hydroxyl groups, so that the ability of reducing Ag^+^ is better than of ethylene glycol. Because the boiling point of glycerol is 290 °C, glycerol as a reaction solution can be heated to over 200 °C, which could improve the reaction speed and promote reduced Ag^+^ to grow Ag nanowires fast. In order to ensure the safety of the equipment, the temperature is controlled below 200 °C. 0.4 M of PVP (*M*_*w*_ ≈ 1,300,000) as a dispersant and capping agent and 0.01 M AgNO_3_ were dissolved in 80 and 20 ml of glycerol, respectively. The viscosity of glycerol and PVP is bigger, so that AgNO_3_ and PVP must be separately dissolved in the glycerol. Then, AgNO_3_ solution added to polyvinylpyrrolidone solution is stirred for 2 min to mix well. The reaction system without other chlorides and template salts could reduce particles and the difficulty of centrifuge separation, so that the purity and yield of Ag nanowires increase. The mixture is transferred into a 100-ml autoclave and heated at 150 °C for 9 h. Autoclave reaction in the oven avoids glyceryl alcohol oxidized by the air to form acrolein and light yellow polymer. After the reaction, the autoclave is cooled in the water, which could terminate Ag nanowire regrowth. On the other hand, it improved the production efficiency. The above all, the synthesis process of silver nanowires is shown in Fig. 1a. Owing to the high viscosity of glycerol and PVP, the products of Ag NWs diluted with acetone 10 times are centrifuged at 2000 rpm/10 min to remove the redundant solvent (glycerol), polyvinylpyrrolidone, and other impurities. After centrifuging, Ag nanowires of uniform length to diameter ratio are dispersed in isopropyl alcohol.

#### Preparation of Ag Nanowire Film on PET

The homogeneous silver nanowires are dispersed in isopropyl alcohol and pumped on the cellulose membrane scattered in isopropyl alcohol for 24 h. Then, cellulose membrane is stacked on PET film though reciprocating rolling process. After that, the edge of cellulose membrane is fixed firmly on the PET film by the scotch tape. And to ensure that silver nanowires and PET film fully combined, PET film is pressed on the hot platen of 150 °C after 10 min. Finally, there will be a cellulose membrane side placed above the acetone solution distillation; cellulose membrane is dissolved to keep silver nanowires on the PET film. Due to the elastic memory effect, PET film is repeatedly pressed on the hot platen of 150 °C to make the combination of silver nanowire intersection fully. So, the flexible transparent conducting thin film is prepared successfully.

### Characterization

The silver nanowires of optical and surface plasmon resonance (SPR) dispersed in alcohol and the transmissivity of thin film are measured using a UV-vis spectrometer in the range of 300–800 nm, which covers the visible light spectrum. The morphology and microstructure of the prepared silver nanowires are characterized by SEM and TEM. XRD pattern for dry silver nanowires studies the crystal structure and chemical composition at a scanning rate of 0.2°/min in the range of 10–80° with Cu *Kα* radiation. PET film roughness is determined using an atomic force microscope (AFM). AFM image exhibits surface morphology of silver nanowires hot-pressed on the PET. Sheet resistance measurements are measured using a digital multimeter. In the end, the uniformity and electrical conductivity film is tested by the closed loop connected to a blue LED.

## Results and Discussion

SEM image of the as-prepared silver nanowires is shown in Fig. 1b. Figure 1b shows nanowires with a uniform length of 10–20 μm and homogeneous diameter of 40–50 nm. The length to diameter ratio is about 400. PVP as dispersing agent and direction of guiding agent is well known to selectively passivate on {100} planes of Ag seeds and leaving the unpassivated {111} planes to grow longitudinally. In the process of the whole test, silver nanowires cannot synthesize without the presence of PVP and the mixed solution is yellow transparent. This result is confirmed and shown in Fig. 1c. The silver nanowires have not only a 5-fold-twinned crystal structure and rounded end shapes, and the twin boundaries are clearly appeared through the entire nanowire. As well known, Ultraviolet-visible absorption of silver nanowires goes along not only the length direction namely longitudinal plasma band but also along the width direction to be called transverse plasma band. UV-vis extinction spectra of silver nanowires scattered in alcohol are shown in Fig. 1d, which indicates SPR peaks at 350 and 380 nm. The peak at ~350 nm is attributed to longitudinal plasma band while other peak is ascribed to transverse plasma band. For further evidence, the crystal structure and crystallinity are analyzed using XRD, which is performed on silver nanowires on glass substrate. As can be seen from Fig. 1e, XRD pattern of silver nanowires is obtained. All peaks of (111), (200), (220), and (311) are indexed to the fcc structure of silver. There are no other peaks that no impurities are detected to indicate the formation of highly pure silver nanowires, which also confirm the raw material without adding any metal halide compound. Therefore, the above results can be proved that this synthesis method can meet the experimental prediction and is very suitable for the preparation of silver nanowires with no impurities.

**Fig. 2:**
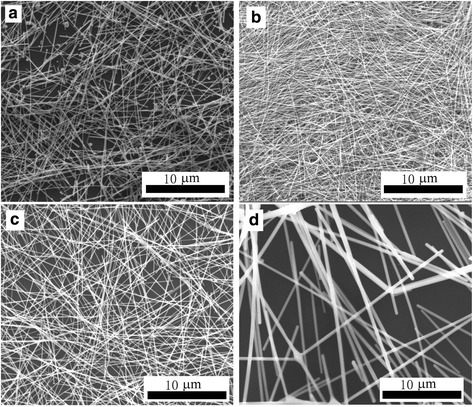
SEM images of silver nanowires synthesized by adjusting the concentration of AgNO_3_ **a** 0.5 times, **b** 1 times, **c** 2 times, and **d** 5 times

To further improve the yield of silver nanowires, it firstly considers increasing the concentration of precursors of AgNO_3_. The results show that aspect ratio of silver nanowires decreases seriously and a large number of nanoparticles as the precursors concentration adjusting, when the other reagents and reaction conditions are same. AgNO_3_ is adjusted 0.5 times, 1 times, 2 times, and 5 times. The obtained silver nanowires are shown in Fig. 2. As can be seen from Fig. 2a, the uniform diameters and lengths of uniform silver nanowires are approximately 40–50 and 10–20 μm, respectively. From Fig. 2b–d, it finds that as the concentration of AgNO_3_ increasing, the aspect ratio of silver nanowires decreases and the clubbed silver wires appear. This would make it to imagine that the concentration of the precursor would increase the aspect ratio of silver nanowires. But the concentration of AgNO_3_ reduces 0.5 times, there are massive nanoparticles and the aspect ratio is not improved. In view of the above information, it is impossible to increase the yield of the silver nanowires from the increase of concentration of the precursor.

**Fig. 3:**
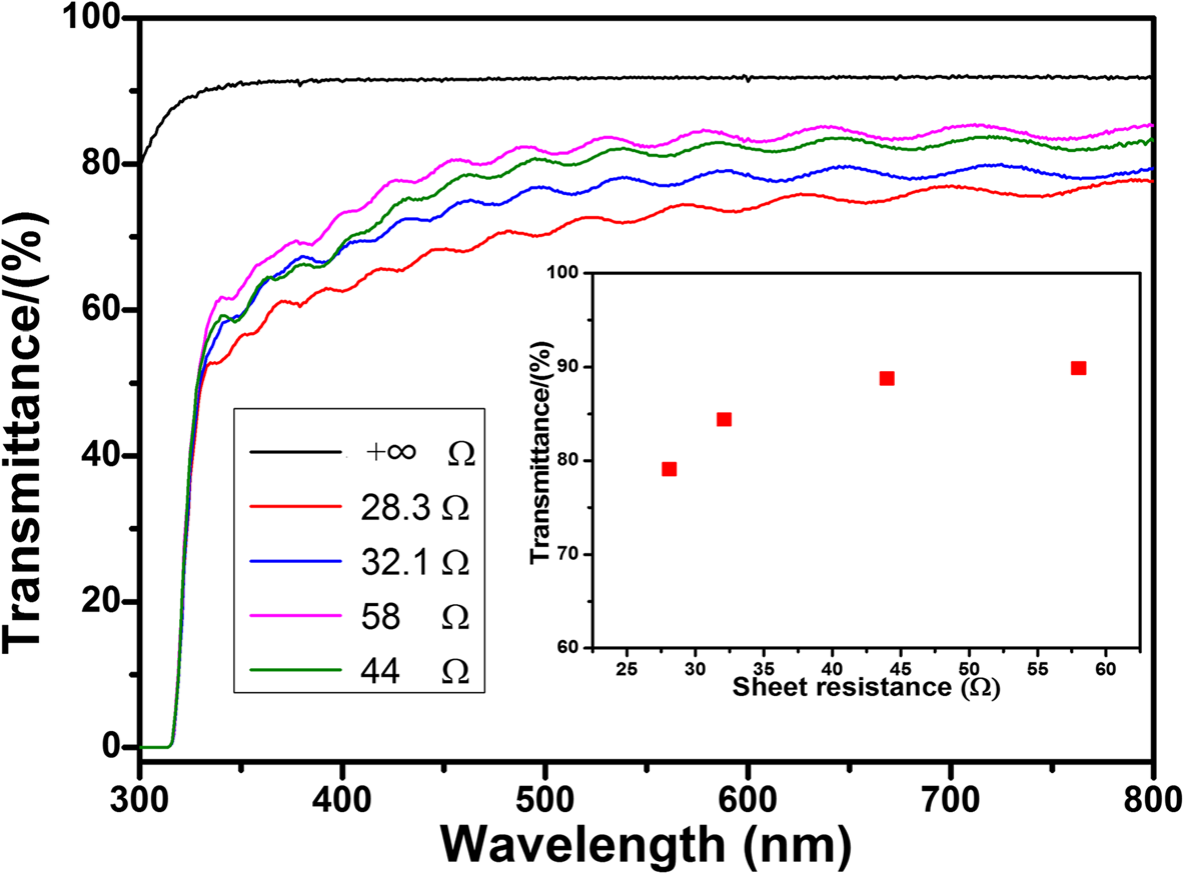
Transmittance vs wavelength plot for thin film by different volumes of Ag nanowire solution, and transmittance vs sheet resistance plot of silver nanowire film with different densities of Ag nanowires

The mechanical properties of flexible transparent conducting thin film are embodied in conductivity and transmittance, which are influenced by aspect ratio and concentration of silver nanowires [11]. Figure 3 shows transmittance vs wavelength plot for thin film and transmittance vs sheet resistance plot of silver nanowire film with different density of the same aspect ratio Ag nanowires. Uniform silver nanowire conductive film is helpful to the stability of the optical and electrical conductivity. As a reference of transmittance, the sheet resistance + ∞ is not suction filter silver nanowires on the PET, whose transmittance is 91.8 at the visible light wavelength of 550 nm. Then, the other transmittance of PET films can be divided by the reference value of 91.8% to obtain the actual transmittance. According to this method, the sheet resistance is 28.3 Ω and transmittance is 79.2. The sheet resistance is 58 Ω and transmittance is 89.9 to catch up with ITO film. Thus, this silver nanowire as a replacement of traditional ITO is becoming possible. The results obviously demonstrate the lower electrical conductivity, the higher the transmittance.

**Fig. 4:**
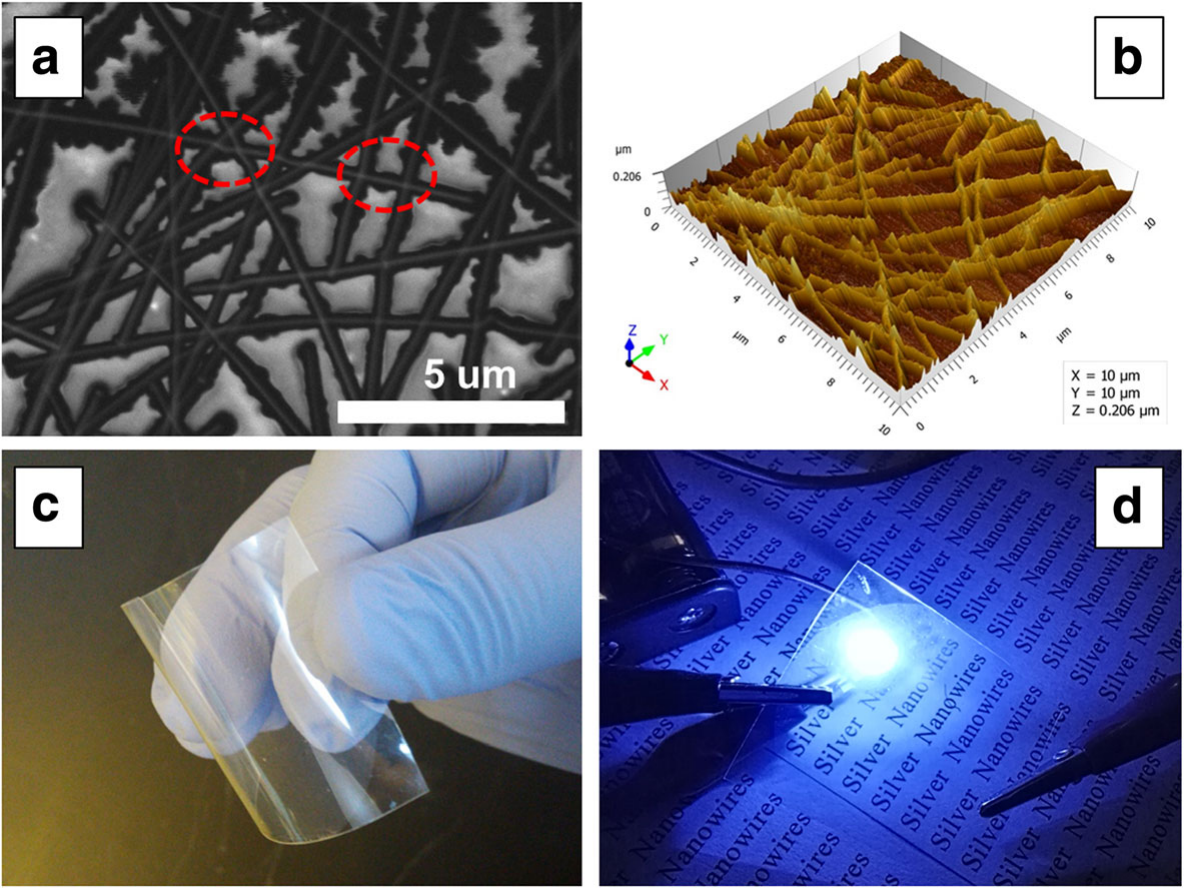
The SEM (**a**) and AFM (**b**) image of the transparent conducting thin film, flexible (**c**), and electron property test (**d**)

The SEM image of the morphology of flexible film is shown in Fig. 4a. It shows the morphology of the PET film with higher magnification and silver nanowires is embedded in the film, and the silver nanowire intersections are fused together. The embedded silver nanowires, due to the suction filter and hot pressing, can be firmly with film laminating and reduce the thickness of the film. The fused intersections of nanowires could improve the conductivity and transmittance of film and also can reduce the roughness of the film. The surface roughness of the AgNW film is 21.1 nm. The AFM picture of PET film is shown in Fig. 4b. AFM image confirms the film contains silver nanowires. The outline of nanowires is clearly visible. And the surface roughness of the film is low. The transparency and electrical conductivity of the film are further detected as shown in Fig. 4c. It shows the flexible transparent film is bended to demonstrate the curved and flexible. In addition, the film can be observed to be transparent with the naked eye. The specific transparency is detected above. The electro-conductibility of the film is detected by a small blue LED, which is connected with a pair of batteries by wires to form a closed loop. LED lights or not to detect the film conducts or not. As shown in Fig. 4d, the LED is lighting in the close loop. This result confirms that the flexible transparency film is conductive.

## Conclusions

In conclusion, we have found a stable synthetic method without any template salt to prepare silver nanowires with controllable aspect ratios on a large scale. The diameters and lengths of nanowires are approximately 40–50 and 10–20 μm, respectively. Without the template, it would increase the stability of experiment and the success rate. And it reduces or eliminates the presence of particles, which derived from silver nanowires that are impossible though other approaches and affect the transmittance, conductivity, and roughness of the film. Pure silver nanowires prepare flexible film, which could keep up with ITO’s transmittance and conductivity. Then, silver nanowires would take place of ITO in the application of the touch screen. It can greatly reduce the cost of the touch screen. Due to the stability of the synthesis and without particles, producing massively silver nanowires becomes possible.
